# Impact of intensity-modulated proton therapy in reducing radiation-induced lymphopenia in glioma patients

**DOI:** 10.1093/noajnl/vdae088

**Published:** 2024-06-04

**Authors:** Anindita Das, Jacinthlyn Sylvia, Ganapathy Krishnan, Pankaj Kumar Panda, Preethi Subramanyam, Roopesh Kumar, Rajendran Adhithyan, Sushama Patil, Dayananda Sharma, Rakesh Jalali

**Affiliations:** Department of Radiation Oncology, Neuro Oncology Cancer Management Team, Apollo Proton Cancer Centre, Chennai, India; Department of Radiation Oncology, Neuro Oncology Cancer Management Team, Apollo Proton Cancer Centre, Chennai, India; Department of Medical Physics, Apollo Proton Cancer Centre, Chennai, India; Department of Clinical Research, Apollo Proton Cancer Centre, Chennai, India; Department of Radiation Oncology, Neuro Oncology Cancer Management Team, Apollo Proton Cancer Centre, Chennai, India; Department of Neurosurgery, Neuro Oncology Cancer Management Team, Apollo Proton Cancer Centre, Chennai, India; Department of Diagnostic & Intervention Radiology, Neuro Oncology Cancer Management Team, Apollo Proton Cancer Centre, Chennai, India; Department of Pathology, Neuro Oncology Cancer Management Team, Apollo Proton Cancer Centre, Chennai, India; Department of Medical Physics, Apollo Proton Cancer Centre, Chennai, India; Department of Radiation Oncology, Neuro Oncology Cancer Management Team, Apollo Proton Cancer Centre, Chennai, India

**Keywords:** gliomas, IMPT, lymphopenia, pencil beam scanning, proton therapy

## Abstract

**Background:**

Current standard management in adult grades 2–4 gliomas includes maximal safe resection followed by adjuvant radiotherapy (RT) and chemotherapy. Radiation-induced lymphopenia (RIL) has been shown to possibly affect treatment outcomes adversely. Proton beam therapy (PBT) may reduce the volume of the normal brain receiving moderate radiation doses, and consequently RIL. Our aim was to evaluate the incidence and severity of RIL during proton beam therapy (PBT).

**Methods:**

We identified patients with grades 2–4 glioma treated with PBT at our center between January 2019 and December 2021. We evaluated the incidence and severity of RIL from weekly complete blood count (CBC) data collected during PBT and compared it to the patients who were treated with photon-based RT (XRT) at our center during the same time.

**Results:**

The incidence of any degree of lymphopenia (48% in PBT, vs. 81.2% in XRT, *P* value = .001) and severe lymphopenia (8% in PBT, vs. 24.6% in XRT, *P* value = .093) were both significantly lesser in patients who received PBT. Severe RIL in patients receiving PBT was seen in only CNS WHO Gr-4 tumors. Mean whole brain V20GyE and V25GyE inversely correlated to nadir ALC and were both significantly lower with PBT. Patients with lymphopenia during PBT showed a trend toward poorer progression-free survival (*P* = .053) compared to those with maintained lymphocyte counts.

**Conclusions:**

Proton therapy seems to have a superior sparing of normal brain to moderate dose radiation than photon-based RT and reduces the incidence of lymphopenia. Glioma patients with lymphopenia possibly have worse outcomes than the ones with maintained lymphocyte counts.

**Key Points**

- Lymphopenia in glioma patients after PBT is less frequent and less severe than after XRT.
- Proton therapy reduces whole brain V20Gy and V25Gy, which are important predictors of lymphopenia.
- Glioma patients with lymphopenia showed a trend toward worse progression-free survival.

**Importance of the Study**

Radiation-induced lymphopenia (RIL) has been linked with poor survival in different cancers, including gliomas. Proton therapy (PBT) is more logistic- and labor-intense than photon-based RT (XRT), hence there is a growing need for evidence to establish any benefits of it over XRT in the management of gliomas, especially the high-grade ones. The present study demonstrates significantly lesser incidence and severity of RIL after PBT, and a trend toward early progression in patients with RIL. High-grade gliomas typically have a poor prognosis despite treatment and hence any intervention that may improve their outcomes is a welcome break and merits further evaluation. Going forward, PBT may make dose-escalation studies more feasible, by reducing toxicities including RIL. Dose-escalation may possibly reduce local failures in HGGs which are the commonest pattern of failure in this group, but is currently often limited by excessive toxicities when delivered by XRT.

Gliomas are the most common primary central nervous system (CNS) tumors, with glioblastoma and grade-4 astrocytoma together accounting for almost half of these.^1^ Following maximal safe resection, the consensus for the management of grades 2–4 gliomas favors a combination of radiation therapy (RT) and chemotherapy, based on consistent benefits seen across several cooperative practice-changing studies including RTOG-9802, RTOG-0424, RTOG-9402, EORTC-26951, and data from an interim analysis of CATNON trial and initial results of the CODEL study.^2–7^ Consequently, this is now adopted into leading clinical practice guidelines, including the latest EANO, ASCO–SNO, and NCCN guidelines and our own experience.^8–12^

Proton beam therapy (PBT), by virtue of its unique physical and radiobiological properties, reduces radiation doses to the organs at risk (OARs), including hippocampi and normal brain. Lesser incidence of RT-related toxicities, fatigue, and long-term neurocognitive decline has been reported in glioma patients after PBT,^11–14^ which is now increasingly being approved as the radiotherapy option of choice for selected LGG patients.^15^ Radiation-induced lymphopenia (RIL) is another well-documented toxicity of RT, both in conjunction and in absence of chemotherapy.^16,17^ The recently published phase-2 trial by Mohan et al. comparing glioblastoma patients treated with photons vs. protons offers an encouraging insight, with significantly lower incidence of severe lymphopenia in the proton arm.^18^ This is an exciting prospect with possible survival connotations, as grade-3 and above RIL after RT in glioma patients, has shown worse median survival and increased risk of death in various studies, reinforced by the pooled data in recent meta-analyses.^19–22^ This detrimental effect of RIL is also repeated across several other cancer types, eg lung, esophageal, pancreatic cancers, and head and neck cancers, where RIL is seen to adversely affect response, disease control, and survival.^23–27^

We report here our early experience of treating grade-2 and above gliomas with proton beam therapy with our focus and emphasis on radiation-induced lymphopenia. Our aim was to explore the incidence of lymphopenia in these patients and compare it with patients treated with photon therapy during the same time at our center, with comparable dose-prescriptions and protocols. We also sought to compare the volume of the whole brain receiving moderate doses, between these 2 groups of patients, and evaluate its effect on lymphopenia, if any.

## Materials and Methods

Between January 2019 and December 2021, all histologically proven CNS WHO grades-2, 3, and 4 gliomas, including patients with both newly diagnosed or recurrent tumors, ie radiation-naïve and reirradiation cases treated with pencil beam scanning-intensity-modulated proton therapy (PBS-IMPT) at our center were included. CNS WHO Gr-1 gliomas such as pilocytic astrocytoma cases were excluded. All cases were treated after discussion in the neuro-oncology multidisciplinary tumor board (MDT). The study was conducted according to the Declaration of Helsinki and the International Conference on Harmonization on Good Medical Practices. Ethics clearance for this retrospective study was obtained from the Institutional Ethics Committee (reference ID APCC202105/APH-C-S-002/02-22).

Demographic details were captured from electronic medical records of patients, available from our clinical workstation, radiology PACS workstation, and Elekta’s Mosaiq^®^ oncology information system. Standard contouring guidelines for gliomas were followed. In this work, we have used the unit Gray-equivalent, abbreviated as GyE, for all dose prescriptions and dosimetric reporting for PBT, in which the relative biological effectiveness (RBE) factor for protons has been accounted for, for a photon-equivalent dose. All PBS–PBT plans were generated using Monte Carlo-based dose optimization and calculation. Both contours and radiation plans were approved by the proton peer-review team, prior to treatment implementation. Dosimetric parameters were recorded from the Treatment Planning System (TPS) Raystation version 11, including the volume of the normal brain receiving 20 GyE and 25 GyE, ie V20GyE, and V25GyE, respectively.

During their radiotherapy treatment, complete blood counts (CBC) data was collected at baseline and thereafter weekly, for all patients, until completion of radiotherapy. Acute toxicities, including lymphopenia, were assessed using NCI CTCAE v5.0. The severity of lymphopenia was accordingly graded as follows, using the absolute lymphocyte count (ALC): Gr-0: ALC > 1000/mm^3^, Gr-1: 801–1000/mm^3^, Gr-2: 501–800/mm^3^, Gr-3:201–500/mm^3^, Gr-4 ≤ 200/mm^3^. Gr-0 was attributed normal, Gr-1 and Gr-2 as mild lymphopenia, and Gr-3 and Gr-4 as severe lymphopenia, clubbed together as Gr-3 + lymphopenia (Gr-3 + L) hereafter. The nadir ALC during treatment and the worst grade lymphopenia were noted for each patient.

We also collected the corresponding data for all consecutive glioma patients treated at our center by photon-based RT (XRT) using intensity-modulated photon therapy (IMRT), during the same period, including demographics, treatment details, weekly CBC data, and whole brain V20Gy and V25Gy, and compared these against the proton therapy cohort. The principles of contouring, dose of RT, Temozolomide prescription, etc. were standard across both groups.

A standard follow-up schedule as per institutional policies was followed for all patients-including the first visit 4 weeks post-RT, and subsequent visits at 3, 6, and thereafter 9 or 12 months. Detailed 1-mm slice thickness plain and contrast-enhanced MRI brain with multi-voxel spectroscopy, dynamic susceptibility contrast (DSC) perfusion, arterial spin labeling (ASL), diffusion-weighted imaging (DWI), and amide proton transfer (APT) sequences were done on follow-up visits. These were then assessed by our neuro-radiologist, discussed in our institutional neuro-oncology MDT, and the findings including response, progression, pseudo-progression, etc. were reported based on the Response Assessment in Neuro-Oncology Criteria for Gliomas (RANO) criteria, supplemented by the advanced imaging sequences. For patients who prefer follow-up at a center closer to their home, the MRI protocol was provided and thereafter the MRI images were obtained remotely in Digital Imaging and Communications in Medicine (DICOM) format, similarly reviewed in our MDT and follow-up records were maintained to the best possible extent. The patients were followed up until September 1, 2022. Progression-free survival (PFS) was defined in our study as the time from commencement of RT, until whenever progression was first detected. Since our cohort contained patients with both new and recurrent tumors, the commencement of RT was selected as the starting point for the calculation of PFS, rather than the date of initial diagnosis. Follow-up details including PFS, details of recurrence, if any, and survival status at the time of data analysis, were captured by a combination of electronic data obtained from our clinical workstation, radiology PACS images and reports, as well as corroborated for updates via suitable telephonic correspondence with the patient’s family, when indicated, to the maximum possible extent. Statistical analysis was performed using IBM SPSS Statistics 22 for Windows (SPSS, Inc., Chicago, IL). Descriptive statistical analysis was applied to routine demographic data collected, expressed as frequency for categorical variables. The chi-square test, *t*-test for independent samples, and Fisher’s exact test were used for comparisons. The Kaplan–Meier method was used to extract PFS with 95% confidence intervals. To assess the correlation between PFS and potentially relevant covariates, the multivariate Cox proportional hazard model was fitted to the data.

## Results

### Demographics

The patient characteristics, including demography, surgical, and chemo-radiation details, are presented in Table 1. We identified a total of 72 patients—50 males and 22 females, with histopathologically confirmed Gr-2, 3, and 4 gliomas who were treated at our center with PBS-IMPT from January 2019 to December 2021. The median age of patients was 40.5 years. Grade-4 tumors were the commonest (44 patients, 61.1%). A total of 69 such patients had been treated with photon-based RT (XRT), delivered by IMRT technique, at our center in the same period. Their details are also presented in Table 1.

**Table 1.** Demographics and Patient Characteristics

Modality of RT Received	Proton Beam Therapy (PBT) (n = 72)	Photon-Based IMRT (XRT) (n = 69)
*Sex*		
Male	50	40
Female	22	29
*Age*		
Median age	40.5 years	54 years
Adults	65	65
Adults > 60 years age	17	19
Children and adolescents	7	4
*Extent of resection*		
GTR	28	30
STR	39	34
Biopsy only	5	5
*Instance of radiation*		
Newly diagnosed: Radiation-naïve	51	59
Recurrent tumors: Reirradiation	21	10
*Histopathology*		
Grade-2	13	13
Astrocytoma	10	11
Oligodendroglioma	3	2
Grade-3	15	12
Astrocytoma	8 (2 PXA)	8
Oligodendroglioma	7	4
Grade-4	44	44
Glioblastoma, NOS, ie “GBM” (IDH-wild)	27	29
Astrocytoma, Grade-4 (IDH-mutant)	5	6
Gliosarcoma	3	2
Epitheloid glioblastoma (BRAFV600E positive)	2	1
Diffuse midline glioma (H3K27M-altered)	6	6
Diffuse hemispheric glioma (H3G34-altered)	1	0
*Concurrent chemotherapy*		
Yes, Cap. Temozolomide	68	62
Yes, others	0	0
No	4	7
*Steroid requirement during RT*		
Yes, ≤2 mg/day	12	19
Yes, >2 mg/day	3	14
No	57	36

Abbreviation: PXA = pleomorphic xantho-astrocytoma.

### Concurrent Chemo-Radiotherapy Details

The initial prescribed dose of proton beam therapy (PBT) ranged from 54 GyE to 59.4 GyE for all these patients (30–33 fractions, in single, 2, or 3 phases), depending on the grade of tumor, location, and proximity to dose-limiting critical OARs (brainstem and chiasm), and prior cranial RT, if any. Standard dose-fractionation of 1.8 GyE per fraction was opted for regardless of the first instance of radiation or reirradiation, however, 4 patients had clinical worsening during the treatment course and required modification to an equivalent hypofractionated, abbreviated schedule for the remaining treatment. Of these 4 patients, 2 patients were receiving radiation for the first time, while 2 were undergoing reirradiation. All other patients tolerated the planned treatment well and completed the entire planned course of PBT.

Sixty-eight (94%) patients also received concurrent Cap. Temozolomide (TMZ) along with PBT, at dose 75 mg/m^2^, ranging from 100 to 160 mg.

### Acute Toxicities During PBT

Sixty-eight (94.4%) patients had Gr-1 dermatitis; 4 (5.6%) patients had Gr-2 dermatitis. No patient had Gr-3 or above dermatitis. In 6 (8%) patients who were on steroids at the start of RT, steroids could eventually be tapered and stopped in less than 2 weeks. Six (8%) patients needed steroids intermittently during RT in low doses within 2 mg/day. Only 3 (4%) patients needed steroids at a dose exceeding 2 mg/day, of which 1 patient also required Inj. Bevacizumab during the final week of PBT, due to significant treatment changes (MGMT methylated). Details of steroid use are presented in Table 1. No other unusual toxicities were noted. Lymphopenia results have been separately reported later in the text.

### Adjuvant Systemic Therapy

Fifty-three (73.6%) of the patients subsequently received sequential, adjuvant Temozolomide. Lomustine (CCNU)-based next-line systemic therapy, ie either single agent CCNU or PCV regimen chemotherapy was prescribed to 6 (8.3%) patients. Other strategies for adjuvant treatment were targeted therapy with ONC-201 (H3K27M altered diffuse midline glioma patients), Inj. Bevacizumab, and observation alone, when indicated.

### Lymphopenia During PBT

**Figure 1:**
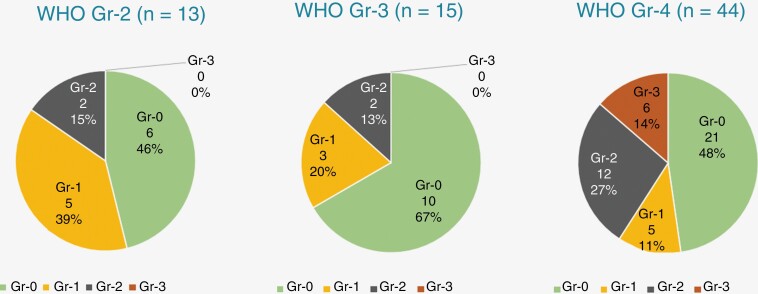
Distribution of lymphopenia grades in different CNS WHO Grade tumors.

The majority of our patients (37 patients, 52%) maintained normal lymphocyte counts through the course of PBT. The remaining 48% had varying grades of lymphopenia; 13 (18%) and 16 (22%) patients respectively had Gr-1 and Gr-2, ie mild lymphopenia, and 6 (8%) patients had Gr-3, ie severe lymphopenia. Interestingly, none of the patients had Gr-4 lymphopenia during PBT. Among the weekly recorded ALCs during treatment, the nadir value was recorded in the 4^th^ and 5^th^ weeks of treatment for a majority of the patients (87.5%, 63 patients). A greater proportion of women had lymphopenia during treatment (44% of men vs. 59.1% of women). However, the rate of Gr-3 + lymphopenia was more in men—10% of men vs. 5% of women. Lymphopenia rates were more in the reirradiation scenario (52%), when compared to the previously radiation-naïve patients (47%). The mean planning target volume (PTV) was 545 ± 201 cm^3^ for the cohort who maintained normal ALC on PBT, and 549 ± 327 cm^3^ for the cohort who had any degree of lymphopenia. The mean PTV between these 2 groups was not significantly different (*P*-value .95, 95% CI –133 to +125). All instances of Gr-3 + L were seen exclusively in the patients with CNS WHO Gr-4 gliomas, and these comprised of 14% of these patients. Moreover, the proportion of patients with any degree of lymphopenia was also higher in CNS WHO Gr-4 cases (52%) vs. in patients with lower-grade tumors (42.9%). Further details of the relative proportion of the lymphopenia grades among patients with CNS WHO Gr-2, 3, and 4 gliomas, are depicted in Figure 1.

Incidence of any degree of lymphopenia, as well as Grade-3 and above lymphopenia, was found to be significantly higher in patients who received XRT, than those who received PBT. Fifty-six (81.2%) of them had varying degrees of lymphopenia, compared to 48% in the PBT patients (*P* < .0001), and 17 (24.6%) had Gr-3 + L, compared to 8% in the PBT patients (*P* = .008). Two of these patients also had Gr-4 lymphopenia vs. none among the PBT patients.

**Figure 2.**
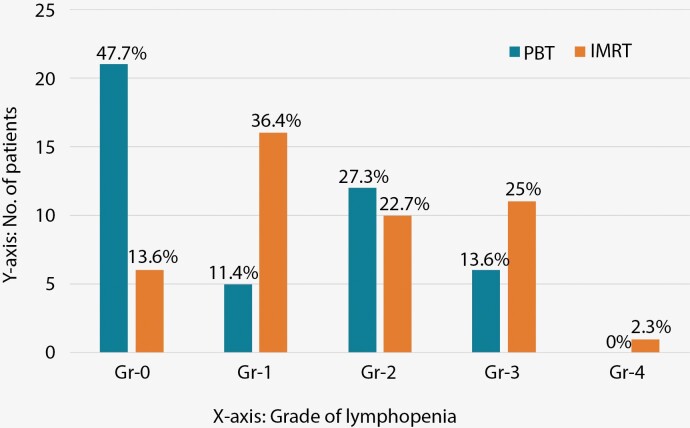
Relative incidence of lymphopenia grades between PBT and IMRT.

Since severe lymphopenia was noted among the PBT patients only when they had CNS WHO Gr-4 gliomas, we compared this cohort further, specifically, with a similar cohort from the IMRT group ie with the patients with CNS WHO Gr-4 tumors (44 patients). Incidence of any lymphopenia in patients with CNS WHO Gr-4 tumors was 52.3% (PBT) vs. 86.4% (XRT) (*P* = .001). Incidence of Gr3 + L was 13.6% (PBT) vs. 27.3% (XRT) (*P* = .093). Mean nadir ALC was higher (931/mm^3^) in the PBT patients compared to the XRT patients (839/ mm^3^). The relative frequency of different grades of lymphopenia in glioblastoma patients after PBT vs. after XRT is compared in Figure 2.

**Figure 3.**
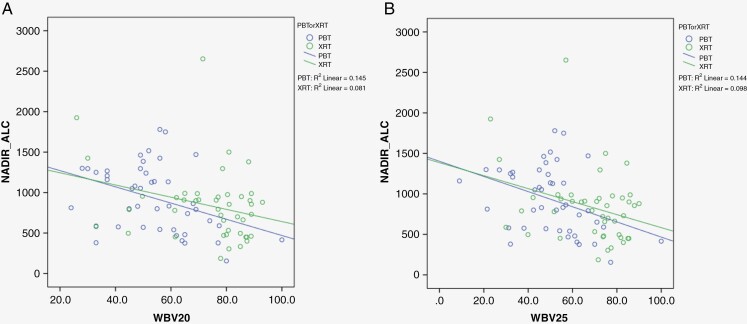
(A) Scatter plots of nadir ALC values for all WHO Gr-4 patients treated with protons (PBT, *n* = 44) and photons (XRT, *n* = 44), plotted against whole-brain V20 Gy. (B) Scatter plots of nadir ALC values for all WHO Gr-4 patients treated with protons (PBT, *n* = 44) and photons (XRT, *n* = 44), plotted against whole-brain V25 Gy. Both whole-brain V20 and V25Gy were negatively associated with the nadir ALC values, ie with an increase in WV_V20Gy and WV_V25Gy, there was worse lymphopenia.

**Figure 4.**
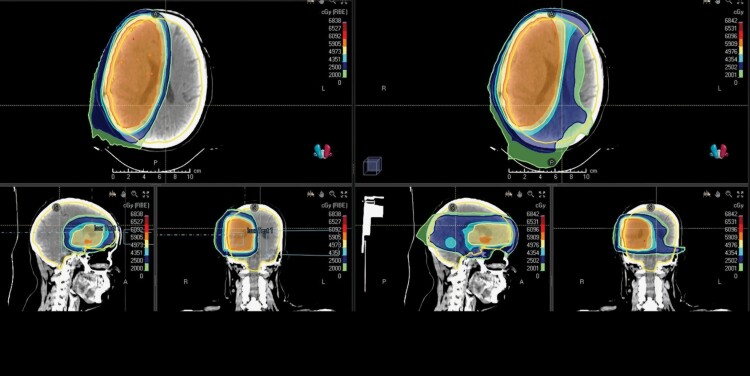

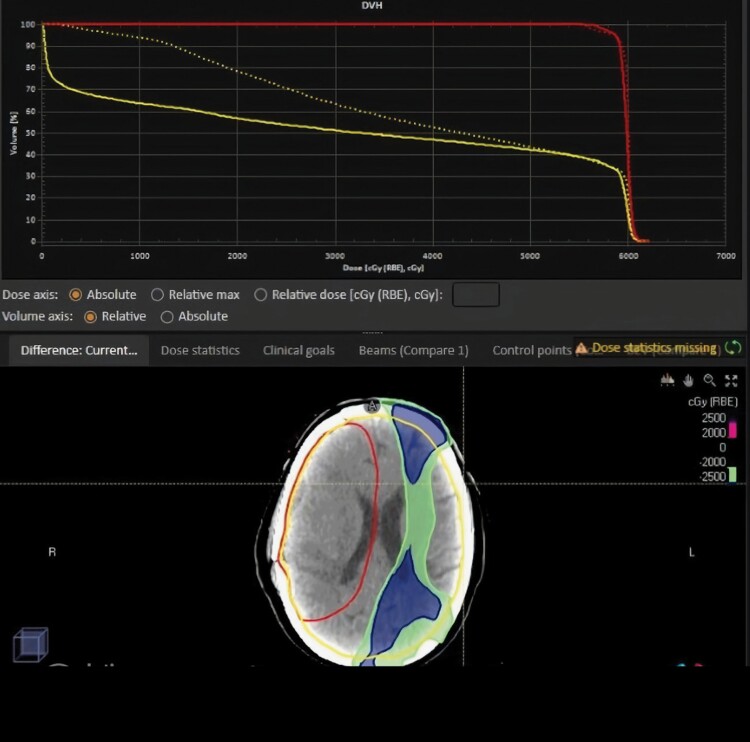
(A) Dose color wash of PBT (IMPT) vs. XRT (IMRT) plans for comparison, in the same patient, showing the moderate dose spill to the normal brain. 20 GyE and 25 GyE coverage are depicted in light green, and Prussian blue, respectively. (B) Relative differences between the PBT and XRT plans are depicted: The DVH shows the whole brain DVH in the the PBT plan (yellow solid line) and in the XRT plan (yellow dotted line); larger whole brain volumes are seen receiving moderate doses in the XRT DVH. Subtracted dose color wash of XRT minus PBT showing the excess areas receiving 20 GyE (light green) and 25 GyE (Prussian blue) in the XRT plan. Both WB V20 GyE and 25 GyE are significantly lesser in the PBT plans.

Whole brain V20GyE and V25GyE were found to be inversely correlated to the nadir ALC values for both PBT and XRT patients, with Pearson *R*-value: 0.409(V20GyE) and 0.412 (V25GyE), *P* < .001. The Pearson scatter correlation plots in Figures 3A, B detail this further. Mean whole brain V20GyE and V25 GyE were both significantly lower in the PBT patients than in XRT patients: Mean WB_V20Gy was 53.9 ± 15.5 cm^3^ and 73.6 ± 16.7 cm^3^, respectively for PBT and XRT patients; *P* < .001. Similarly, mean WB_V25Gy was 50.3 ± 16.6 cm^3^ and 68.0 ± 17.2 cm^3^, respectively for PBT and XRT patients; *P* < .001. Figure 4A shows a dose color wash for the PBT and XRT plans for the same patient and Figure 4B shows the difference between the whole brain DVH between the PBT and XRT plans, as well as the relative subtracted dose wash—that is, the excess volume of the normal brain outside the target that received 20 Gy and 25 Gy doses in the PBT plan, but not in the XRT plan.

### Follow-Up and Survival

Follow-up duration ranged from 6 to 40 months; the median time to progression after completion of PBT for all patients was 12 months. As expected, patients with CNS WHO Gr-4 tumors had more instances of progression. Median PFS for patients with CNS Gr-2 and -3 tumors was not reached, while patients with CNS WHO Gr-4 tumors had a median PFS of 10 months. The log-rank (Mantel–Cox) test showed significant differences among the survival curves (*P* < .001, refer to Supplementary Material for figure) between the patients with Grade-2 vs. Grade-3 vs. Grade-4 tumors. Most of the recurrences were within the treated field; 50% (14) patients had recurrence only within the treated field, while another 32.1% (9) had recurred both within the treated field as well as elsewhere in the brain or leptomeninges. The remaining 17.9% (5) patients recurred outside the radiation field only. Treatment at recurrence was tailored as per the patient’s general condition, previous treatment, time from previous RT, and histopathology including molecular tests. The most common treatments at recurrence were repeat surgery, reirradiation, second-line chemotherapy (Lomustine-based or Bevacizumab–Irinotecan), targeted therapy in case of specific mutations (eg dual BRAF inhibitors) or best supportive care only.

**Figure 5.**
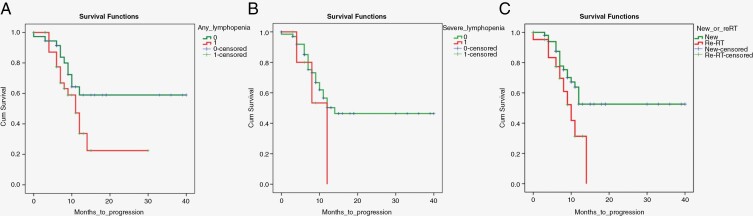
(A) Kaplan–Meier curve of PFS in patients with any lymphopenia vs. normal ALC (*P* = .053-Log-rank, Mantel–Cox). (B) Kaplan–Meier curve of PFS in patients with severe lymphopenia vs. no or mild lymphopenia (*P* = .29). (c) Kaplan–Meier curve of PFS between newly diagnosed patients vs. patients who received reirradiation (ReRT) (*P* = .05).

The median time to progression for patients correlated to lymphopenia during treatment. Patients with no lymphopenia during treatment were found to fare better the median PFS in these patients was not reached, as more than 50% of them had not progressed at the time of the study. On the contrary, the median PFS for patients with any degree of lymphopenia was 11 months, and that for patients with severe lymphopenia was 8 months. The Kaplan–Meier survival analysis curve plotted for patients with no lymphopenia vs. any degree of lymphopenia (Figure 5A) showed a noticeable separation of the survival curves between patients without any lymphopenia during treatment, when compared to those with any degree of treatment-induced lymphopenia, with the former showing better PFS. The log-rank (Mantel–Cox) test was found to be approximating significance (*P* = .053). The Kaplan–Meier survival curve plotted for patients with severe (Gr-3 + L) lymphopenia vs. those with no or mild lymphopenia (Gr-0 to 2) (Figure 5B) also showed separating curves in favor of longer times to progression in the latter; however, this curve had an unequal survival distribution (*P* = .29) since there were only 6 instances of Gr-3 + L in our patients after PBT. However, notably, 50% of these patients who developed Gr3 + L had progressed, and that too with a short median time to progression of 6 months only. There was no significant difference in the rates of RIL between patients who were newly diagnosed and receiving RT for the first time, vs. patients receiving reirradiation. However, as expected, the latter subgroup progressed sooner posttreatment, and the survival curves in Figure 5C elucidate this (*P* = .05).

## Discussion

We have demonstrated lesser prevalence and severity of lymphopenia during adjuvant concurrent chemoirradiation in CNS WHO Gr-2 and above glioma patients treated with proton beam therapy. Proton therapy, by virtue of its physical characteristics like rapid dose falloff and resultant superior dosimetry, reduced normal brain exposure and hence was more immune-forgiving and caused less lymphopenia when compared to modern, intensity-modulated, photon-based RT. Patients who received proton therapy had a longer time to progress, raising hope for a potential survival advantage secondary to this lymphocyte-sparing effect, given the dismal survival in glioblastomas and other Grade-4 tumors otherwise, even in the era of modern oncology and its many advances.

Although the main focus of this study was to report lymphopenia in patients during PBT, we did have the advantage of having a corresponding XRT arm for comparison, since many patients were also contemporarily treated with IMRT at our center following the same, standardized, peer-reviewed treatment principles, under the same multidisciplinary neuro-oncology cancer management team. The choice of modality for delivering RT ie PBT vs. XRT, was discussed extensively and clearly with every patient and/or caregiver, including extensive discussion on what the anticipated magnitude of benefit with PBT tailored to that specific patient would be—negligible, limited, modest, or significant. The final decision was guided by patient preference and their logistical feasibility, including self-funding, insurance support, etc., given the larger healthcare cost associated with PBT.

To our best knowledge, ours is the only other study apart from the phase-2 randomized trial by Mohan et. al., MD Anderson Cancer Centre, to have compared lymphopenia in gliomas in a proportionate number of patients treated with protons versus photons.^18^ While the quoted study had 28 and 56 patients with glioblastoma in the PBT and XRT arms, respectively, ours compared a total of 72 and 69 patients with Gr 2–4 gliomas, in the PBT and XRT arm, respectively, of which 44 patients each in PBT and XRT arms had glioblastoma.

All the patients treated with either proton therapy or photon therapy at our center, had histological confirmation prior to radiotherapy, either after maximal safe resection, or biopsy. Thereafter, they received adjuvant RT with concurrent oral Temozolomide (TMZ), followed sequentially by further oral TMZ for 6–12 cycles. The treatment paradigm essentially followed the established Stupp protocol in glioblastoma.^28^ Although rigid conformity to the Stupp protocol mandates a dose-fractionation of 2 GyE per fraction for glioblastomas, a dose-fractionation ranging between 1.8 and 2 GyE is practiced widely for the treatment of high-grade gliomas including glioblastomas and recommended across guidelines.^8,10^ Our favored dose-fractionation for gliomas has also been 1.8 GyE per fraction as radiobiological models predict biological iso-efficacy while potentially reducing late toxicities like radiation necrosis and neurocognitive decline. Although there is still an ongoing debate regarding adjuvant PCV (Procarbazine, CCNU/ Lomustine, and Vincristine) vs. TMZ chemotherapy, especially for oligodendrogliomas (ODG), high incidence of dose delays, dose reduction, and premature discontinuation of chemotherapy is commonly noted with PCV.^4,5^ The contrasting favorable safety profile and ease of administering have made TMZ emerge as the advocated option; parallelly a head-to-head comparison of these is now underway, in the amended CODEL protocol.^29^

Proton beam therapy was well tolerated in our patients along with concurrent Cap. Temozolomide, with no patient suffering from any acute, radiation-induced, grade-3 or above toxicity. Except for 4 patients with poor general condition either unwilling or unfit for Temozolomide, all the other patients, regardless of whether newly diagnosed or previously treated, received concurrent Cap Temozolomide during PBT due to its radio-sensitizing potential.

Interestingly, the need for continued steroid use during RT was minimal in our patients receiving PBT- with a small proportion of patients needing low-dose steroids < 2 mg/day and even fewer patients needing a dose > 2mg/day, beyond 2 weeks during ongoing RT. This may bear a positive consequence since prolonged and/or high-dose use of Dexamethasone has both been shown in several reports to adversely affect lymphocyte counts and also overall survival in glioblastoma patients.^30,31^ The XRT group showed a higher requirement of steroids during treatment, which could have also contributed to the development of lymphopenia during RT and have partially added to the higher incidence and severity of the same in this group, compared to the ones who received PBT.

From the retrospective analysis of the prospectively collected CBC parameters during RT, we found that in all Gr-2 and above gliomas, as well as specifically in Gr-4 gliomas, there was better immune sparing with PBT. The overall incidence of any degree of lymphopenia and acute severe lymphopenia, ie Gr-3 + L, were both significantly lower, and mean nadir ALC was higher, in the patients who received PBT than those who received XRT. In fact, among the patients who received PBT, Gr-3 + L was only found in the CNS WHO Gr-4 patients, again in keeping with the immune evasive strategies seen in glioblastoma.^32^ The incidence of lymphopenia in our patients with glioblastoma after PBT was comparable to that reported by Mohan et al. (13.6% vs. 14% Mohan et. al.).^18^ The incidence of lymphopenia in our comparison-XRT group also paralleled those from literature (27.3% vs. 27.6%, Zhang et al.^21^).

Our results also showed that higher whole-brain V20GyE and V25 GyE both correlated to more severe lymphopenia and lower nadir ALC values, across both PBT and XRT patients. Both whole-brain V20GyE and V25GyE were again significantly lower in the patients who received PBT, compared to those who received XRT. This is consistent with prior reports where larger volumes of the normal brain being exposed to low to moderate doses of radiation have been implicated to cause worse lymphopenia—particularly whole brain V20Gy and V25Gy, which have been repeatedly found to be some of the strongest dosimetric predictors of severity of lymphopenia.^18,19,33^ Our results provide evidence to support the hypothesis that proton therapy, by virtue of its ability to restrict low and medium-dose spills, can reduce RIL.

Patients with any degree of lymphopenia in our study showed a trend towards worse progression-free survival and shorter time to progression when compared to the ones who maintained normal ALC values during treatment. Further, follow-up is required to arrive at more robust data on progression and overall survival. An analysis of the US National Cancer Database (170 PBT vs. 49 405 XRT), had shown superior median and 5-year survival in glioma patients treated with PBT than XRT: 45.9 vs. 29.7 months (*P* = .009) and 46.1 vs. 35.5% (*P* = .0160), respectively.^34^ The exciting new developments in terms of several reports now showing that radiation-induced lymphopenia translates to worse response and recurrence rates and poorer survival across several cancer types,^23–27^ including gliomas,^19–22,35^ can potentially be exploited. Corollary, with interventions directed at maintaining better lymphocyte counts, there is a possibility of bettering treatment outcomes. Historically, glioblastomas have been found to have exaggerated lymphopenia compared to lower-grade gliomas. The usual immune evasive actions of glioblastoma, including a propensity to reduce T-cell lymphocyte counts, give rise to further indirect evidence that lymphopenia may directly be involved in adversely affecting outcomes and effects of treatment may be augmented by countering the same.^32,36^

Reduced exposure of normal brain irradiated to low and medium doses—particularly whole brain V20GyE and V25GyE, reduced exposure of lymphoid tissue ie the CNS lymphatics around the dural venous sinuses, and smaller treatment volumes, have all been correlated to reduced lymphopenia.^33,37,38^ This study opens a scope for some interesting and more directed research. Considering the direct correlation between whole brain exposure and RIL, further research can help understand if adopting WB_V20GyE and WB_B25GyE as actual dose-constraints while planning can help further reduce lymphopenia. Although traditionally the brain was considered an immune-privileged site, newer research over the last 2 decades has demonstrated the presence of a meningeal lymphatic network, with conventional lymphatics following the dural venous sinuses and exiting the CNS near the base of the skull.^39^ Since proton beam therapy can also achieve better dose sculpting, in future, therefore, it may be worthwhile to consider the CNS lymphatic-rich areas as newer OARs, and more specific constraints may be prescribed to avoid these areas, while planning the beam geometry and assessing the treatment plans. Optimal research to investigate the potential survival benefits of PBT in glioma management is still lacking. We hope to get some answers regarding this from the ongoing NRG-BN001 trial comparing PBT vs. IMPT for newly diagnosed glioblastoma patients, which will also answer whether dose escalation may likely be beneficial in glioblastomas.^40^ If so, PBT can help in further widening the therapeutic ratio. For Grades-2 and -3 IDH-mutant gliomas, the ongoing NRG-BN005 is a randomized phase-2 trial investigating long-term cognitive outcomes and quality of life between PBT and IMRT patients receiving standard dose-RT.^41^ Further research in this regard should also integrate the lymphoid-sparing directed constraints as described above, to investigate any effects of integrating it prospectively in the planning process itself.

The main limitations of this study arise due to the retrospective design. Since the PBT and XRT arms are not actual prospectively randomized groups, bias arising from confounding factors which are not obvious, cannot be discounted. Some apparent ones include the imbalanced age and sex distribution of patients between these groups. The XRT group consisted of more women, and although the proportion of elderly patients, ie older than 60 years in both the groups was nearly equal, overall, the XRT patients had an older median age. Women and elderly patients both being groups where RIL is known to be more prevalent, this could have thus added to the higher burden of lymphopenia in the XRT group. However, our results also showed that although the women in our study were more likely to have lymphopenia, severe lymphopenia was seen more often among the male population and therefore the sex distribution alone does not explain the higher occurrence of lymphopenia, particularly of severe lymphopenia, in the XRT population much more than the PBT one. The higher use of Dexamethasone in the XRT group also means that it could independently contribute to the worse lymphopenia seen in this group, however, this can also be interpreted as an advantageous effect of PBT by reducing steroid-dependence, further supporting its cause for use in selected patients who may benefit from it. The relatively short follow-up period also limited us from reporting more robust survival data.

## Conclusions

In conclusion, our results show a lower incidence of treatment-induced lymphopenia during radiotherapy with proton therapy, compared to modern, photon-based IMRT, along with concurrent Cap Temozolomide. A higher volume of the whole brain receiving 20 GyE and 25 GyE was also significantly correlated to worse lymphopenia and reaffirms these parameters as important predictors of acute severe lymphopenia. Notably, proton therapy was found to reduce the normal brain V20GyE and V25GyE, therefore significantly reducing lymphopenia. Patients with lymphopenia showed a trend towards faster progression, therefore emphasizing the need for further studies in this regard. The findings of this study should be considered hypothesis-generating, and dedicated prospective studies with continued follow-up should be considered, to evaluate the role of modern, intensity-modulated proton therapy, in reducing treatment-induced lymphopenia and survival.

## Supplementary Material

vdae088_suppl_Supplementary_Figure
