# Supplementary material for: Impact of intensity-modulated proton therapy in reducing radiation-induced lymphopenia in glioma patients
Source: Neurooncol Adv. 2024 Jun 4;6(1):vdae088. doi: 10.1093/noajnl/vdae088 (PMC11263926; doi:10.1093/noajnl/vdae088)

Supplementary figure: Kaplan Meier curve of PFS between patients with CNS WHO Grade-2, Grade-3, and Grade-4 gliomas showing successively shorter time to progression with increasing tumour grade (p < 0.001)


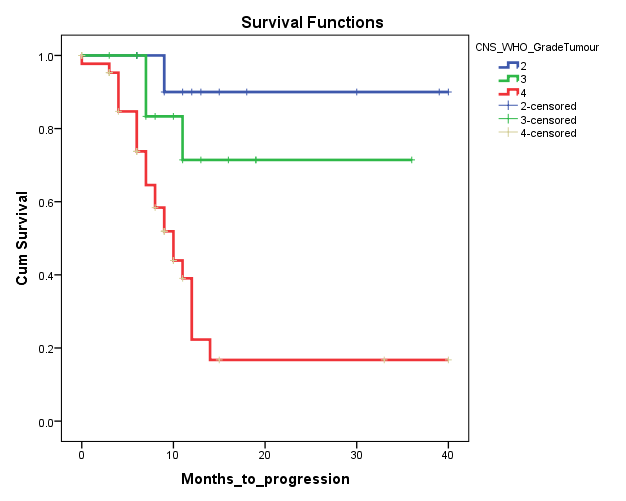

Supplement: vdae088_suppl_Supplementary_Figure [file vdae088_suppl_supplementary_figure.docx]
